# A Fungal-Derived Bioactive Resource for Cochlear Protection: *Sanghuangporus sanghuang* Extract Mitigates Acoustic Trauma through Nrf2/HO-1 Antioxidant Axis

**DOI:** 10.4014/jmb.2605.05011

**Published:** 2026-06-08

**Authors:** Hojin Lee, Changho Lee, Yun-Tai Kim, JaeYong Park, Jaekwang Lee

**Affiliations:** 1 Food Functionality Research Division, Korea Food Research Institute, Wanju 55365, Republic of Korea; 2 School of Biosystems and Biomedical Sciences, College of Health Sciences, Korea University, Seoul 02841, Republic of Korea; 3 Major in Food Biotechnology, University of Science and Technology, Daejeon 34113, Republic of Korea

**Keywords:** Noise-induced hearing loss, *Sanghuangporus sanghuang*, Nrf2/HO-1 signaling, Oxidative stress, Otoprotection

## Abstract

Noise-induced hearing loss (NIHL) is a major form of sensorineural hearing impairment driven by oxidative stress–mediated cochlear injury. *Sanghuangporus sanghuang* (SS), a medicinal fungus extensively studied in microbiology and biotechnology, is known to produce bioactive metabolites with antioxidant properties; however, its functional role in the auditory system has not been established. This study investigated the otoprotective potential of SS extract against oxidative and acoustic stress using complementary *in vitro*, ex vivo, and *in vivo* models. In H_2_O_2_-treated UB-OC1 auditory cells, SS (25–200 μg/mL) dose-dependently restored cell viability and significantly reduced intracellular reactive oxygen species accumulation. Western blot analysis demonstrated that SS suppressed the expression of apoptotic markers, including cleaved caspase-3 and cytochrome C. At the molecular level, SS upregulated Nrf2 and HO-1 expression at both mRNA and protein levels, without a significant change in Keap1 expression, indicating activation of endogenous antioxidant defense via the Nrf2/HO-1 signaling axis. Consequently, downstream antioxidant genes such as SOD1 and NQO1 were significantly upregulated. In *ex vivo* cochlear explant cultures, SS preserved hair cell integrity against H_2_O_2_-induced damage, as confirmed by phalloidin staining. In a murine NIHL model, oral administration of SS attenuated ABR threshold shifts, maintained Wave I amplitudes, and preserved the structural organization of outer hair cells across all cochlear turns. Collectively, these findings demonstrate that SS confers otoprotection by modulating the Nrf2/HO-1 antioxidant axis and mitigating oxidative stress–induced sensory cell injury, supporting the potential of SS as a fungal-derived functional bioactive resource for redox-associated cochlear protection.

## Introduction

Noise-induced hearing loss (NIHL) is the second most common form of sensorineural hearing loss worldwide, after age-related hearing loss, affecting an estimated 5% of the global population and imposing substantial individual and societal burdens [1, 2]. Despite widespread awareness of its preventable nature, NIHL remains a major public health concern, highlighting the need for effective nutritional strategies and functional dietary components to support auditory health [3, 4]. The cochlea is exceptionally vulnerable to acoustic trauma due to its high metabolic demand and limited regenerative capacity. Exposure to intense noise triggers rapid and sustained generation of reactive oxygen species (ROS), including superoxide anion and hydroxyl radicals, in cochlear hair cells and supporting tissues [5, 6]. This oxidative overload disrupts redox homeostasis, promotes lipid peroxidation — reflected by accumulation of cytotoxic aldehydes such as 4-hydroxynonenal (4-HNE) — damages mitochondrial integrity, and ultimately activates the intrinsic apoptotic cascade, including release of cytochrome c and activation of cleaved caspase-3, leading to irreversible loss of sensory outer hair cells (OHCs) [7, 8]. OHCs, particularly in the basal turn of the cochlea, are preferentially vulnerable owing to their lower intrinsic antioxidant reserves, making them the primary cellular target of noise-induced oxidative injury [7, 9, 10].

Endogenous cochlear antioxidant systems — encompassing superoxide dismutase (SOD), catalase, and glutathione peroxidase — provide a first line of defense against ROS; however, these systems are readily overwhelmed under conditions of intense acoustic challenge [11]. Nuclear factor erythroid 2–related factor 2 (Nrf2) serves as the master transcriptional regulator of cellular antioxidant and cytoprotective programs [12]. Under basal conditions, Nrf2 is sequestered in the cytoplasm by its repressor, Kelch-like ECH-associated protein 1 (Keap1), and targeted for proteasomal degradation. Upon oxidative activation, Nrf2 dissociates from Keap1, translocates to the nucleus, and binds antioxidant response elements (AREs) to induce downstream effectors including heme oxygenase-1 (HO-1), NAD(P)H quinone oxidoreductase 1 (NQO1), and SOD1 [13-15]. Critically, Nrf2 is expressed in inner and outer hair cells, supporting cells, and the stria vascularis of both human and murine cochleae [16, 17]. Nrf2-deficient mice exhibit accelerated auditory threshold elevation and exaggerated hair cell loss following noise exposure, whereas Nrf2 activation by bioactive compounds prior to acoustic trauma attenuates permanent threshold shifts and preserves hair cell integrity — indicating that Nrf2-mediated pathways are crucial for the protective efficacy of bioactive compounds against noise-induced oxidative stress [18, 19]. Consistent with this, several natural polyphenolic compounds — including rosmarinic acid and caffeic acid — have been shown to amplify cochlear Nrf2/HO-1 signaling and reduce oxidative cochlear damage following noise exposure [19, 20].

This evidence base has spurred considerable interest in food-derived bioactive compounds with established safety profiles as candidate otoprotective functional ingredients [3, 21]. *Sanghuangporus sanghuang* (syn. *Phellinus linteus*, also known as Meshimakobu), a perennial medicinal fungus belonging to the family Hymenochaetaceae, has been used for over two millennia in East Asian traditional medicine and is of substantial biotechnological interest for its diverse functional metabolites [22, 23]. Bioactive constituents of SS include polysaccharides (notably β-glucans), polyphenolic styrylpyrones (including hispidin and its dimers), triterpenoids, and phenylpropanoids — a chemical profile associated with broad bioactivities encompassing antioxidant, anti-inflammatory, immunomodulatory, hepatoprotective, and neuroprotective effects [22-24]. At the cellular level, hispidin, a representative phenolic compound of SS, has been demonstrated to activate Nrf2/HO-1 signaling, suppress intracellular ROS, and inhibit caspase-3–mediated apoptosis in diverse oxidative stress models [25, 26]. Furthermore, SS polysaccharides have been shown to activate the AMPK/Nrf2 pathway and attenuate oxidative injury in hepatocyte models [27]. These findings collectively suggest that SS–derived bioactive compounds possess the molecular prerequisites for modulating the cochlear Nrf2 antioxidant axis. Despite this promising bioactive profile and the well-established role of the Nrf2/HO-1 axis in cochlear protection, whether SS-derived constituents engage this antioxidant pathway in auditory sensory cells has not been examined.

Based on these lines of evidence, we hypothesized that SS extract would confer otoprotection by actively engaging the Nrf2/HO-1 antioxidant axis in auditory sensory cells. To test this hypothesis, we employed a multi-tiered experimental approach comprising *in vitro* experiments using UB-OC1 auditory cells, ex vivo organotypic cochlear explant cultures, and an *in vivo* murine model of acoustic trauma. By evaluating SS across cellular viability, ROS modulation, apoptotic signaling, Nrf2 pathway activation, and functional auditory outcomes, this study provides mechanistic and functional evidence highlighting the potential of SS as a functional bioactive resource for maintaining redox homeostasis in auditory cell models.

## Materials and Methods

### Preparation of SS Extract

Dried *Sanghuangporus sanghuang* fruiting bodies were obtained from a commercial producer (Jirisan Sanghuangbogam, Saengbiryang-myeon, Sancheong-gun, Gyeongnam Province, Republic of Korea; harvest date: October 2024). The dried SS material is a commercially available product; representative morphological documentation of the dried fruiting body can be verified through the supplier's product information. The raw material was pulverized using a blender to yield a homogeneous dry powder. A total of 180 g of the resulting dried powder (dry weight basis) was used as the starting material for extraction. Extraction was carried out using a 10-fold volume of solvent (1.8 L) of 70% ethanol. The extraction solvent was prepared by diluting food-grade fermented ethanol (Jungju Pankye World Co., Ltd., Republic of Korea) with distilled water to a final concentration of 70% (v/v). The 70% EtOH solvent system was specifically selected because it preferentially co-extracts polar phenolic constituents — including hispidin, caffeic acid, and protocatechuic acid — alongside amphiphilic β-glucan–protein conjugates that collectively account for the major Nrf2-activating and antioxidant activities attributed to SS extracts [23, 24]. The extraction was conducted at 80°C for 6 h with constant rotation at 110 rpm. Following extraction, the solution was filtered through Whatman No. 4 filter paper under vacuum, concentrated by rotary evaporation (60°C water bath), and lyophilized at -70°C for 72 h. The lyophilized extract was stored at -20°C until use. From 180 g of dry material, 17.96 g of extract was obtained, corresponding to an extraction yield of 9.97% (w/w).

### Cell Culture and Differentiation

UB-OC1 cells (#CVCL-9636, XIMBIO) were cultured and differentiated according to the protocol previously described in our study [28]. Briefly, cells were maintained in Dulbecco’s Modified Eagle’s Medium (DMEM; Gibco, USA) supplemented with 10% fetal bovine serum (FBS) and 1% penicillin/streptomycin. For the proliferation phase, cells were incubated at 33°C with 5% CO_2_ in the presence of 5 μl/mL IFN-γ for 8 days. To induce differentiation, the medium was replaced with IFN-γ-free DMEM, and the cells were further incubated at 39°C for 13 days. Experiments were performed using cells between passages 8 and 15, maintained at 37°C and 5% CO_2_. To ensure reproducibility, all assays were conducted in three to five independent experiments, with each condition performed in triplicate.

### Cell Viability Assay

Cell viability was assessed via the MTT assay as previously described [28]. Briefly, cells in 96-well plates were treated with 700 μM H_2_O_2_ with or without SS for 24 h. After 3 h of incubation with MTT solution (5 mg/mL), the formed formazan was dissolved in DMSO. Absorbance was recorded at 570 nm. Data were obtained from four independent replicates (n = 4).

### Intracellular ROS Measurement and Imaging

Intracellular ROS levels were evaluated as previously described [28]. Briefly, UB-OC1 cells were treated with 700 μM H_2_O_2_ in the presence or absence of SS or NAC (5 mM) for 24 h. Following treatment, cells were stained with DCFDA (Invitrogen, USA), and the fluorescence intensity was measured using a microplate reader at excitation and emission wavelengths of 485 nm and 535 nm, respectively. To visualize the intracellular distribution of ROS, cells were labeled with CellROX™ Green Reagent (Invitrogen). Representative fluorescence images were captured using a confocal laser scanning microscope (FV3000, Olympus, Japan) at 20× and 60× magnification. The relative fluorescence intensity was quantified across multiple randomly selected fields of view using FV31S-SW software (Olympus). All assays were performed in at least three independent experiments to ensure statistical reproducibility.

### Western Blotting

Total proteins were extracted from UB-OC1 cells using a lysis buffer and quantified using a BCA protein assay kit. Equal amounts of protein were separated by SDS-PAGE and subsequently transferred onto PVDF membranes (Bio Rad, USA). Following blocking with 5% non-fat milk, the membranes were incubated overnight at 4°C with specific primary antibodies (1:1000). The membranes were then washed and incubated with HRP-conjugated secondary antibodies (1:5000) for 1 h at room temperature. Protein bands were visualized using an enhanced chemiluminescence (ECL) imaging system, and the band densities were quantified using ImageJ software (NIH). All blots were obtained from at least three independent experiments, and the relative protein expression was normalized to β-actin to ensure data consistency and reproducibility. All antibodies used in this study are listed in Supplementary Table S2.

### qRT-PCR

Total RNA was extracted using the NucleoSpin RNA Kit (Macherey-Nagel, Germany) and reverse-transcribed into cDNA using RT Master Mix (TOYOBO, Japan) according to the manufacturer’s instructions. Quantitative PCR was performed on a QuantStudio™ 6 Flex Real-Time PCR System (Thermo Fisher Scientific, USA) using SYBR Green (TOYOBO). The relative expression levels of target genes were normalized to β-actin and calculated using the 2^-ΔΔ Ct^ method. All primer sequences used in this study are provided in Supplementary Table 2. Data were obtained from at least three independent experiments.

### Immunocytochemistry (ICC)

Immunocytochemistry was performed to visualize the expression and subcellular localization of target proteins, following the protocols previously established in our study [28]. UB-OC1 cells were cultured on poly-D-lysine–coated coverslips in 12-well plates and subjected to 700 μM H_2_O_2_ treatment for 24 h, either in the presence or absence of SS (50, 100, 200 μg/mL) and NAC (5 mM). Following treatment, cells were fixed with 4% paraformaldehyde (PFA) for 15 min at room temperature and subsequently permeabilized and blocked with a solution containing 0.3% Triton X-100 and 5% normal serum in PBS for 1 h. The cells were then incubated overnight at 4°C with specific primary antibodies. After washing with PBS, cells were labeled with Alexa Fluor™ 488 or 555-conjugated secondary antibodies (Abcam, UK) for 1 h at room temperature in the dark. Fluorescence images were captured using a confocal laser scanning microscope (FV3000, Olympus) at 20× and 60× magnification. The relative fluorescence intensity was quantified across multiple randomly selected fields of view using FV31S-SW software. To ensure statistical reliability, all imaging and quantification procedures were repeated in at least three independent trials. Detailed information regarding the antibodies used is summarized in Supplementary Table S2.

### *Ex vivo* Cochlear Explant Culture and Hair Cell Staining

Cochlear explant cultures were performed as previously described [28] with minor modifications. Cochleae were microdissected from postnatal day 5 (P5) C57BL/6 mice. The organ of Corti, including the sensory epithelium, was isolated in ice-cold Hank’s Balanced Salt Solution (HBSS). The explants were then placed on MatTek dishes pre-coated with Cell-Tak (Corning, USA) and NaHCO_3_ for 1 h to allow for tissue attachment. The cultures were maintained in 2 mL of cochlear culture medium at 37°C in a humidified atmosphere of 5% CO_2_. After a 24-h stabilization period, the explants were treated with 100 μM H_2_O_2_ alone or in combination with 200 μg/mL SS for an additional 24 h. Following treatment, the explants were fixed in 4% paraformaldehyde (PFA) and blocked with a blocking solution. To visualize the hair cell bundles, the tissues were stained with Alexa Fluor™ 488 Phalloidin (1:400) according to the manufacturer’s protocol. Fluorescence images were acquired using a confocal laser scanning microscope (FV3000, Olympus). The fluorescence intensity of Phalloidin was quantified using ImageJ software (NIH, USA). To ensure statistical reproducibility, all results were obtained from at least three independent explant cultures.

### Animals Care and Oral Administration of SS

**Fig. 1:**
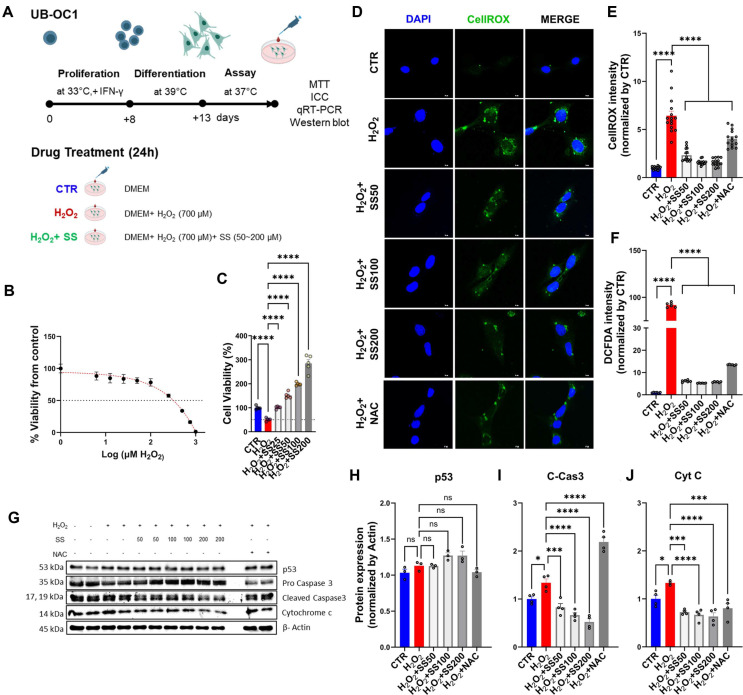
SS-mediated cytoprotection and inhibition of ROS accumulation in H_2_O_2_ treated UB-OC1 cells. (**A**) Schematic diagram of the experimental timeline for UB-OC1 cell proliferation, differentiation, and drug treatment. (**B**) Dose-response curve of UB-OC1 cell viability following H_2_O_2_ treatment (0–1000 μM) for 24 h. (**C**) Cytoprotective effect of SS extract (25–200 μg/mL) on H_2_O_2_ (700 μM) induced cytotoxicity measured by MTT assay. (**D–F**) Representative images and quantitative analysis of intracellular ROS levels using CellROX (green) and DCFDA staining. NAC (5 mM) was used as a positive antioxidant control. (**G–J**) Representative Western blot bands and densitometric analysis of apoptosis-related proteins, including p53, Cleaved Caspase-3 (C-Cas3), and Cytochrome c (Cyt C). β-Actin was used as an internal loading control. Data are presented as mean ± SEM from at least three independent experiments. *p < 0.05, ***p < 0.001, ****p < 0.0001.

All animal procedures were approved by the KFRI Institutional Animal Care and Use Committee (KFRI-M-23018). Six-week-old male C57BL/6 mice were maintained under controlled conditions (20–24°C, 12 h light/dark) with ad libitum food and water. Mice were randomly assigned to Sham, Noise, or Noise+SS groups (n = 5–6/group). Oral administration was selected because it is the clinically and practically relevant route for food-derived bioactive compounds, enabling direct translation to dietary supplementation strategies, and is consistent with our prior validated otoprotection studies [21, 28]. The SS extract, dissolved in saline, was administered orally at 200 mg/kg/day for 14 consecutive days; Sham and Noise groups received equivalent saline volumes. The dose of 200 mg/kg/day was selected using the following three-tier rationale: (i) *in vitro* MTT assays confirmed >90% cell viability at 200 μg/mL (Fig. 1A and 1B), establishing the safe and effective cellular concentration boundary, consistent with non-cytotoxic ranges of 25–400 μg/mL for SS-related extracts in published studies [22, 23]; (ii) applying FDA BSA normalization using Km correction factors (mouse *K*_m_ = 3, 60-kg human *K*_m_ = 37; FDA Guidance for Industry, CDER, 2005 [44]), this dose corresponds to an HED of approximately 16.2 mg/kg/day (~0.97 g/day for a 60-kg adult; ~1.13 g/day for a 70-kg adult), within the empirically established effective oral dose range of SS extracts in murine models (100–400 mg/kg [22]) and consistent with the 1–2 g/day range in traditional and commercial use [33]; acute oral safety is confirmed at >18.2 g/kg in mice (~2,724 × human daily dose) [7]; and (iii) this dose is identical to that validated in our prior buckwheat [21] and sorghum [28] otoprotection studies in the same model, providing direct dose-equivalency precedent. The 14-day pretreatment ensured sustained systemic exposure prior to acoustic challenge [21, 28].

### Auditory Assessment and Noise Exposure

Prior to noise exposure, baseline auditory brainstem response (ABR) thresholds were recorded. Mice were anesthetized with 2% Avertin (0.02 mL/g, i.p.) and maintained on a 37°C heating pad. ABRs were measured in response to click stimuli (90–20 dB SPL, 5 dB steps) using a Tucker-Davis Technologies system. Following baseline recordings, mice were exposed to 110 dB SPL white noise (2–20 kHz) for 6 h per day over 3 consecutive days. Exposure was conducted in soundproof cages equipped with calibrated overhead speakers (InterM) to ensure uniform sound distribution. Post-exposure ABR thresholds were measured using the same parameters. The degree of hearing loss was quantified by calculating the threshold shift (post-exposure threshold minus baseline threshold) for each mouse. Additionally, the amplitude and latency of Wave I (measured from the peak to the following trough) was analyzed to evaluate functional changes in the auditory nerve.

### Cochlear Immunohistochemistry and Whole-Mount Preparation

Mice were anesthetized and transcardially perfused with phosphate-buffered saline (PBS) followed by 4% paraformaldehyde (PFA). The cochleae were dissected and post-fixed in 4% PFA for 24 h at 4°C. Subsequently, the samples were decalcified in 0.5 M EDTA (pH 8.0) for 3 days at 4°C. The decalcified cochleae were microdissected into apical, medial, and basal turns. The isolated tissues were blocked and permeabilized with 0.3% Triton X-100 and 10% normal serum in PBS. To visualize the integrity of hair cells, the tissues were incubated with Alexa Fluor™ 488 Phalloidin (1:400) for 1 h at room temperature. The samples were then mounted with DAPI-containing Fluoroshield (Sigma-Aldrich, USA) to counterstain the nuclei. For cross-sectional analysis, decalcified cochleae were cryoprotected in 30% sucrose overnight and embedded in OCT compound. The tissues were sectioned at a thickness of 15 μm using a cryostat (Leica, Germany). The sections were blocked and incubated overnight at 4°C with anti-4HNE (1:1000). Following PBS washes, the sections were labeled with an Alexa Fluor™ 555 secondary antibody (1:1000) and Phalloidin. All fluorescence images were acquired using a confocal laser scanning microscope (FV3000, Olympus). The fluorescence intensity of 4-HNE was quantified using FW software (Olympus) by measuring the mean gray value within the organ of Corti regions of interest (ROIs) of the cochlear turns. For each experimental group, at least three mice (n = 3) were utilized, and all imaging and quantification procedures were repeated in three independent trials to ensure reproducibility. Detailed information regarding the primary and secondary antibodies, as well as other reagents used in this study, is provided in Supplementary Table 2.

### Statistical Analysis

All experimental procedures and data assessments were conducted under randomized and blinded conditions. Data processing and statistical evaluations were performed using GraphPad Prism 10 (GraphPad Software, USA). All results are presented as the mean ± standard error of the mean (SEM), derived from at least three independent biological replicates. For comparisons between two groups, Student’s t-test was employed. For multiple group comparisons, a one-way analysis of variance (ANOVA) was performed, followed by Tukey’s post-hoc test for pairwise comparisons. A *p*-value < 0.05 was considered to indicate a statistically significant difference.

## Results

### SS Reduces Oxidative Stress–Induced ROS Accumulation and Apoptotic Cell Death in UB-OC1 Auditory Cells

To evaluate the cytoprotective potential of SS against oxidative insult, UB-OC1 auditory cells were differentiated through sequential proliferation at 33°C with IFN-γ and subsequent differentiation at 39°C, as illustrated in the experimental workflow (Fig. 1A). Cells were then challenged with H_2_O_2_ (700 μM, 24 h) in the presence or absence of SS (50–200 μg/mL) or N-acetylcysteine (NAC, 5 mM) as a positive control. The H_2_O_2_ dose–response curve confirmed a concentration-dependent reduction in cell viability, and 700 μM was selected as the challenge concentration for all subsequent experiments (Fig. 1B).

Prior to bioactivity evaluation, HPLC analysis of the 70% ethanol SS extract was performed to characterize its phenolic constituents. Caffeic acid and hispidin were detected and confirmed by co-elution with authenticated reference standards (Supplementary Fig. 1S; Supplementary Table S1). MTT assay demonstrated that SS dose-dependently restored cell viability under H_2_O_2_ challenge, with all tested concentrations (SS 25, 50, 100, and 200 μg/mL) achieving statistically significant improvements relative to the H_2_O_2_-only group (*p* < 0.0001 for all), and the higher concentrations reaching levels comparable to the untreated control (Fig. 1C). Intracellular ROS accumulation was assessed by two independent fluorescent probes, CellROX and DCFDA. H_2_O_2_ treatment produced an increase in fluorescence intensity with both probes relative to the untreated control (Fig. 1D–1F). SS co-treatment significantly suppressed ROS elevation: CellROX fluorescence was significantly reduced at SS 100 μg/mL (*p* < 0.0001) and SS 200 μg/mL (*p* < 0.0001), with the H_2_O_2_+NAC positive control also showing a significant reduction (*p* < 0.0001) (Fig. 1E). DCFDA fluorescence was similarly and significantly attenuated at SS 100 μg/mL (*p* < 0.0001) and SS 200 μg/mL (*p* < 0.0001) (Fig. 1F). Confocal imaging of CellROX-stained cells confirmed dose-dependent attenuation of the intracellular oxidative signal in SS-treated groups compared to the H_2_O_2_-only condition (Fig. 1D).

To determine whether SS suppresses downstream apoptotic execution, Western blot analysis was performed for p53, cleaved caspase-3 (C-Cas3), and cytochrome c (Cyt C) (Fig. 1G-1J). p53 protein expression did not differ significantly from the H_2_O_2_-only group at any SS concentration tested (ns for all groups; Fig. 1H), indicating that SS does not substantially alter p53-dependent stress signaling at the protein level under these conditions. In contrast, cleaved caspase-3 was significantly and dose-dependently reduced by SS: SS 50 μg/mL, SS 100 μg/mL, SS 200 μg/mL, and H_2_O_2_+NAC all showed statistically significant suppression relative to the H_2_O_2_-only group (Fig. 1I). Similarly, cytochrome c expression was significantly decreased at SS 50 μg/mL, SS 100 μg/mL, SS 200 μg/mL, and H_2_O_2_+NAC, demonstrating that SS suppresses the upstream mitochondrial apoptotic cascade across the tested concentration range (Fig. 1J). Collectively, these results demonstrate that SS effectively attenuates intracellular ROS accumulation and inhibits mitochondrial apoptotic signaling in oxidatively stressed UB-OC1 auditory cells.

### SS Activates the Nrf2/HO-1 Antioxidant Signaling Axis through Enhanced Nuclear Translocation of Nrf2

**Fig. 2:**
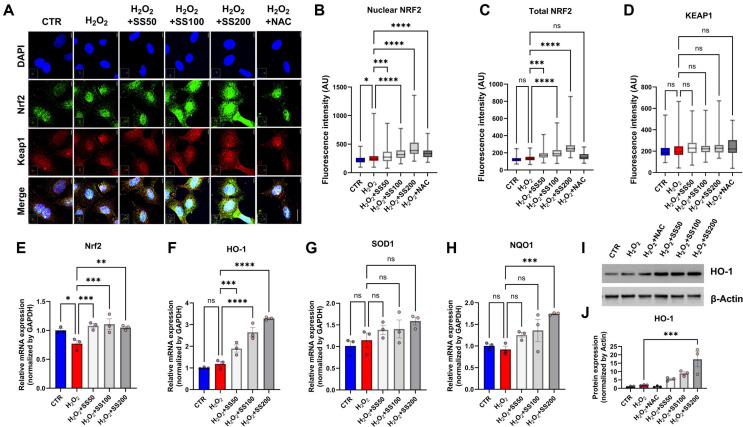
Nrf2 nuclear translocation and upregulation of HO-1 expression by SS in UB-OC1 cells. (**A**) Representative confocal images showing the immunofluorescence of Nrf2 (green) and Keap1 (red) in UB-OC1 cells. Nuclei were counterstained with DAPI (blue). (**B–D**) Quantitative analysis of fluorescence intensity for nuclear Nrf2, total Nrf2, and Keap1. (**E–H**) Relative mRNA expression levels of antioxidant-related genes (Nrf2, HO-1, SOD1, and NQO1) measured by qRT-PCR and normalized to β-Actin. (**I, J**) Representative Western blot image and quantitative analysis of HO-1 protein expression normalized to β-Actin. Data are expressed as mean ± SEM. *p < 0.05, **p < 0.01, ***p < 0.001, ****p < 0.0001.

Given that the Nrf2/HO-1 axis is a principal transcriptional regulator of cellular antioxidant defense in cochlear sensory cells, we next examined whether SS-mediated ROS suppression is mechanistically linked to activation of this pathway by analyzing the effect of SS on Nrf2 nuclear translocation and downstream antioxidant gene expression by immunocytochemistry (ICC), quantitative RT-PCR (qRT-PCR), and Western blot analysis (Fig. 2).

ICC analysis of UB-OC1 cells stained with antibodies against Nrf2, Keap1, and DAPI revealed that SS induced a dose-dependent increase in nuclear Nrf2 fluorescence intensity (Fig. 2A and 2B). All SS concentrations produced statistically significant increases in nuclear Nrf2 compared to the H_2_O_2_-only group: SS 50 μg/mL, SS 100 μg/mL, SS 200 μg/mL, and H_2_O_2_+NAC (Fig. 2B). Total cellular Nrf2 fluorescence was also significantly elevated at SS 50 μg/mL, SS 100 μg/mL, and SS 200 μg/mL , while H_2_O_2_+NAC showed a non-significant trend (ns; Fig. 2C). Importantly, Keap1 fluorescence intensity remained unchanged across all treatment groups (ns for all comparisons vs. H_2_O_2_; Fig. 2D), indicating that SS promotes Nrf2 nuclear accumulation without measurably reducing total Keap1 protein abundance at the immunofluorescence level. This pattern suggests that SS-derived bioactive compounds may facilitate Nrf2 dissociation from Keap1 or enhance Nrf2 protein stability through post-translational mechanisms, rather than through transcriptional or proteasomal suppression of Keap1.

At the transcriptional level, qRT-PCR analysis demonstrated that Nrf2 mRNA was significantly upregulated in a dose-dependent manner: SS 50 μg/mL, SS 100 μg/mL, and SS 200 μg/mL (Fig. 2E). HO-1 mRNA was dose-dependently elevated at SS 50 μg/mL, SS 100 μg/mL, and SS 200 μg/mL (Fig. 2F). In contrast, SOD1 mRNA expression showed no statistically significant change at any SS concentration (ns for all; Fig. 2G), and NQO1 mRNA was significantly elevated only at the highest tested concentration (SS 200 μg/mL), with lower doses having no significant effect (Fig. 2H). These findings indicate that HO-1 is the primary Nrf2-downstream antioxidant gene induced by SS in UB-OC1 cells, with NQO1 responding only at higher concentrations and SOD1 remaining unaffected under the current experimental conditions.

At the protein level, Western blot analysis confirmed a significant and dose-dependent increase in HO-1 protein expression, with SS 200 μg/mL achieving a statistically significant elevation compared to the H_2_O_2_-only group (Fig. 2I and 2J), consistent with the transcriptional data. Taken together, these findings demonstrate that SS activates the Nrf2/HO-1 signaling axis through enhancement of Nrf2 nuclear translocation and consequent transcriptional induction of HO-1, thereby reinforcing endogenous antioxidant defense in auditory cells under oxidative stress.

Having established that SS activates the Nrf2/HO-1 antioxidant axis in UB-OC1 cells (Fig. 2A–2J), we next examined whether hispidin, a phenolic constituent detected in the SS extract by HPLC (Supplementary Fig. S1; Supplementary Table S1), may contribute to this activity. Hispidin was selected on the basis that caffeic acid — also detected in the extract — showed minimal Nrf2/HO-1 activity in UB-OC1 cells in our prior study [28], and that hispidin has been reported to activate the Nrf2/HO-1 axis [25, 26, 29]. Purified hispidin (1–20 μM) dose-dependently attenuated H_2_O_2_-induced cytotoxicity (Supplementary Fig. S2) and suppressed ROS accumulation (Supplementary Fig. S3) in UB-OC1 cells, consistent with a partial contribution to the cytoprotective effects of the SS extract. The potential contributions of other uncharacterized SS constituents remain to be resolved through future fractionation studies.

### SS Preserves Cochlear Hair Cell Integrity against Oxidative Damage in *ex vivo* Organotypic Cochlear Explants

**Fig. 3:**
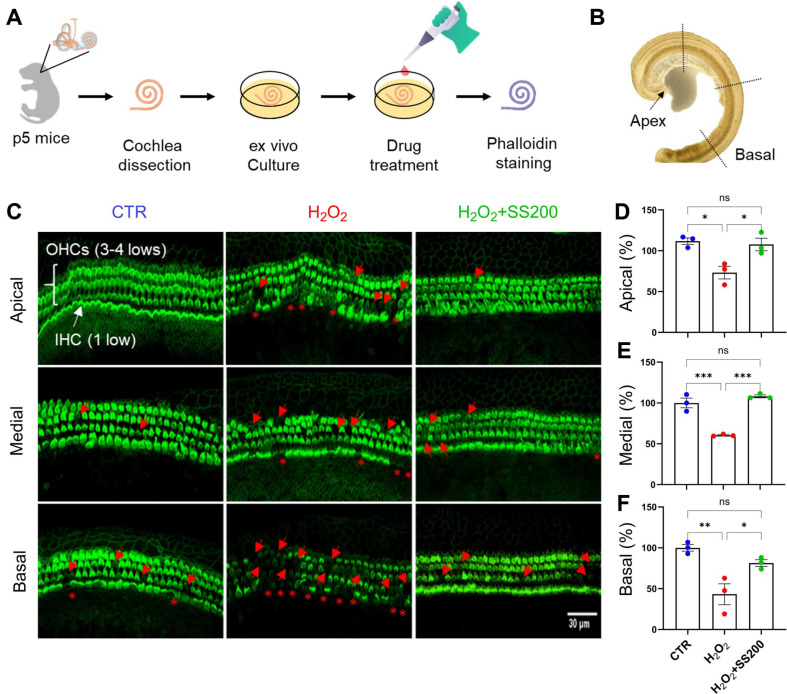
Protective effects of SS against H_2_O_2_ -induced hair cell death in *ex vivo* cultures. (**A**) Schematic illustration of the *ex vivo* cochlear explant culture and drug treatment procedure using P5 mice. (**B**) Representative image of a dissected cochlea showing the apex, medial, and basal turns. (**C**) Representative confocal images of Phalloidin-stained (green) hair cells in the apical, medial, and basal turns following CTR, H_2_O_2_, and H_2_O_2_+SS200 treatment. Red arrowheads (OHCs) and asterisk (IHCs) indicate missing hair cells. (**D–F**) Quantitative analysis of hair cell survival (%) in each cochlear turn normalized to CTR. Scale bar = 30 μm. Data are presented as mean ± SEM (n = 3 mice per group). *p < 0.05, **p < 0.01, ***p < 0.001.

To extend the *in vitro* findings to a structurally intact, multicellular cochlear tissue context, we employed an organotypic *ex vivo* explant model. Organs of Corti were dissected from P5 C57BL/6 mice, maintained in culture, and challenged with H_2_O_2_ in the presence or absence of SS (200 μg/mL), as illustrated in the experimental workflow (Fig. 3A). The cochlear duct was partitioned into apical, medial, and basal segments to enable tonotopic analysis, and hair cell survival was quantified from phalloidin-stained confocal images as percent hair cell loss relative to the untreated control (Fig. 3B).

In the apical segment, H_2_O_2_ treatment significantly increased hair cell loss compared to the untreated control , and SS co-treatment significantly reduced this loss, restoring hair cell counts toward control levels; notably, the difference between the SS-treated group and the untreated control did not reach significance (ns), indicating substantial protection in this tonotopic region (Fig. 3C and 3D). In the medial segment, H_2_O_2_-induced hair cell loss was more pronounced , and SS conferred significant and robust protection, substantially restoring hair cell survival (Fig. 3C and 3E). In the basal segment — the cochlear region preferentially vulnerable to oxidative injury due to its high-frequency tuning and elevated metabolic demands — H_2_O_2_ produced significant hair cell loss, which was significantly attenuated by SS (Fig. 3C and 3F). Across all three cochlear regions, SS-treated explants did not differ significantly from the untreated control (ns for all), demonstrating consistent protection across all tonotopic regions.

Confocal images confirmed that H_2_O_2_-treated explants exhibited disruption of the OHC mosaic, with prominent gaps in the three to four outer hair cell rows and scattered loss of inner hair cells (IHCs, denoted by red arrowheads in the images), most extensively in the medial and basal turns. In contrast, SS-treated explants preserved the organized arrangement of OHCs (3–4 rows) and the single IHC row across all cochlear turns, closely resembling the untreated control (Fig. 3C-3F). These *ex vivo* results confirm that SS directly protects cochlear sensory epithelial integrity in a native multicellular tissue microenvironment, and that this protection extends consistently across the entire tonotopic axis.

### Oral Administration of SS Attenuates Noise-Induced Auditory Dysfunction *in vivo*

**Fig. 4:**
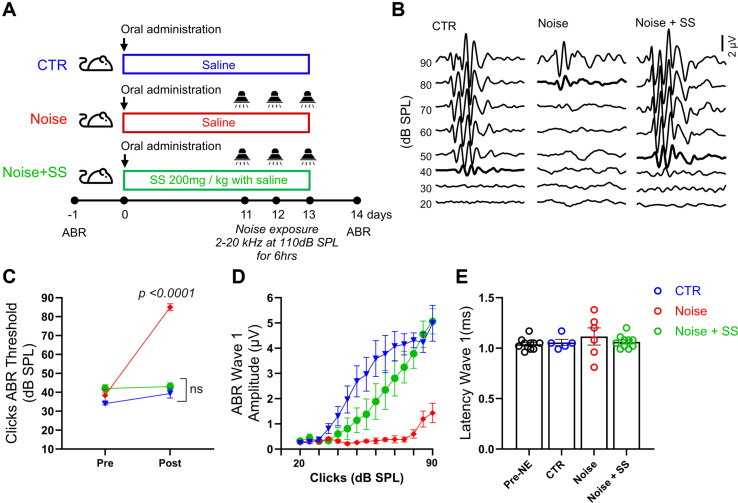
Effect of SS on noise-induced hearing loss (NIHL) and oxidative stress in mice. (**A**) Experimental timeline of oral administration (Saline or SS 200 mg/kg), noise exposure (110 dB SPL, 6 h/day for 3 days), and ABR measurements. (**B**) Representative ABR waveforms at 24 h (day 14) post-noise exposure. (**C**) Comparison of Click-ABR thresholds before (Pre) and after (Post) noise exposure. (**D**) ABR Wave I amplitude input-output functions in response to click stimuli. (**E**) Latency of ABR Wave I at 90 dB SPL. Data are presented as mean ± SEM. For ABR data (B–E), n = 5–6 mice per group. ***p < 0.001, ****p < 0.0001.

To evaluate the *in vivo* otoprotective relevance of SS, we used a C57BL/6 murine model of acoustic trauma. As illustrated in the experimental timeline (Fig. 4A), mice received oral administration of SS (200 mg/kg/day in saline) for 14 consecutive days; noise exposure (2–20 kHz broadband noise, 110 dB SPL, 6 h/day) was applied on days 11, 12, and 13, and ABR recordings were obtained both at baseline (day −1) and 24 hours after the final noise exposure (day 14).

Representative click-evoked ABR waveforms demonstrated that the Noise group exhibited significantly attenuated and flattened wave morphology across nearly all stimulus intensities (20–90 dB SPL), consistent with severe acoustic injury and loss of synchronized auditory nerve firing (Fig. 4B). In contrast, the Noise+SS group retained well-differentiated ABR waveforms with preserved peak morphology at 40-90 dB SPLs, more closely resembling the CTR group (Fig. 4B-4D).

Wave I latency did not differ significantly among the pre-noise exposure (Pre-NE), CTR, Noise, and Noise+SS groups (Fig. 4E), indicating that SS did not alter the conduction velocity of the auditory pathway and that group differences in ABR threshold and amplitude are attributable to sensory rather than neural conduction changes.

Quantitative analysis of click ABR thresholds showed that the Noise group exhibited a significant post-exposure threshold elevation relative to its own pre-exposure baseline (*p* < 0.0001), with mean thresholds rising to approximately 80 dB SPL (Fig. 4C). In contrast, the Noise+SS group maintained post-exposure thresholds that were not significantly different from pre-exposure values (ns), with thresholds remaining at approximately 40–45 dB SPL — a level comparable to that of the CTR group (Fig. 4C). ABR Wave I amplitude input-output functions across the full stimulus range (20–90 dB SPL) showed that Noise mice exhibited severely and consistently reduced amplitudes at all tested intensities compared to CTR animals (Fig. 4D). The Noise+SS group, however, maintained amplitude profiles higher than the Noise group and more closely approximating those of the CTR group across the tested intensity range, indicating preservation of auditory nerve fiber recruitment capacity.

### Oral Administration of SS Suppresses Cochlear Lipid Peroxidation and Ameliorates Noise-Induced Cochlear Damage in Mice

**Fig. 5:**
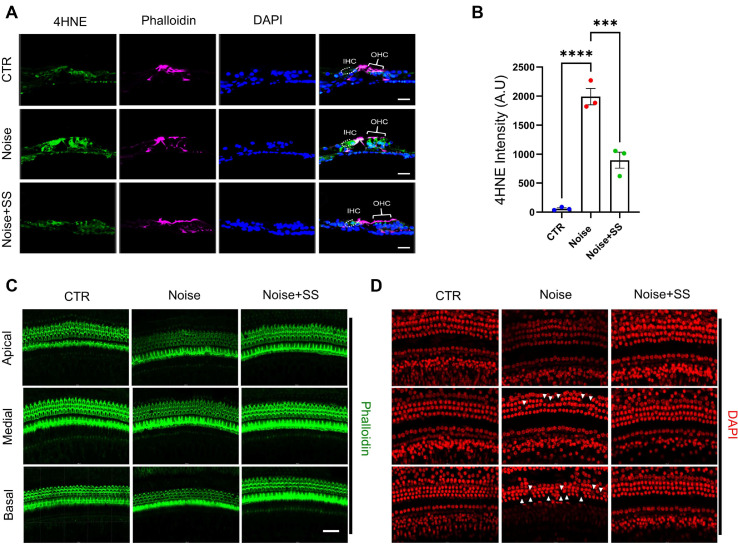
SS treatment preserves the structural integrity of the organ of Corti in noise-exposed mice. (**A, B**) Representative immunofluorescence images (**A**) and quantitative analysis (**B**) of 4-HNE (green) expression in the organ of Corti. Phalloidin (magenta) and DAPI (blue) were used to visualize hair cells and nuclei, respectively. Scale bar = 20 μm. (**C, D**) Representative confocal images of the apical, medial, and basal turns of the cochlea showing Phalloidin (green) (**C**) and DAPI (red) (**D**) staining. In Noise group, white arrowheads indicate disordered or missing nuclei and stereocilia bundles. SS intake contributed to maintaining the structural organization of the organ of Corti in the presence of acoustic challenge. Scale bar = 40 μm. Data are presented as mean ± SEM (n = 3 mice per group). ***p < 0.001, ****p < 0.0001.

To establish a structural and biochemical correlate for the functional hearing protection described above, we examined cochlear tissue changes following noise exposure. Given that acoustic trauma drives oxidative injury and subsequent sensory cell loss, we first performed immunofluorescence staining for 4-hydroxynonenal (4-HNE) to assess cochlear lipid peroxidation (Fig. 5A).

In Noise mice, intense 4-HNE immunoreactivity was observed in both the IHC and OHC regions of the organ of Corti, whereas CTR mice showed minimal 4-HNE signal. Quantitative densitometry confirmed that 4-HNE fluorescence intensity was significantly elevated in the Noise group compared to CTR group (*p* < 0.0001; Fig. 5B). SS treatment significantly reduced 4-HNE immunoreactivity relative to the Noise group (*p* < 0.001; Fig. 5B), demonstrating that oral SS administration effectively suppresses lipid peroxidation in the cochlear sensory epithelium following acoustic trauma. Together, these functional and biochemical findings indicate that oral SS administration attenuated noise-induced cochlear injury in this animal model.

Next, to evaluate the structural integrity of the organ of Corti, cochlear whole-mount preparations were examined by confocal microscopy following phalloidin staining (actin cytoskeleton of hair cell stereocilia and cuticular plates; green channel, Fig. 5C) and DAPI staining (nuclei; red channel, Fig. 5D) across the apical, medial, and basal turns in CTR, Noise, and SS-treated animals.

In the Noise group, DAPI staining (Red; Fig. 5D) clearly demonstrated a significant loss of OHC nuclei across the medial and basal segments, providing direct evidence of irreversible cellular degeneration in these high-frequency regions. Correspondingly, phalloidin imaging (Green; Fig. 5C) revealed a reduction in fluorescence intensity throughout all cochlear turns. While the stereotypical three-to-four-row OHC mosaic pattern was still discernible, the F-actin signal was notably faint and inconsistent, particularly in the regions where nuclear loss was confirmed. In the apical turn, although OHC density was slightly reduced compared to the CTR group, the degenerative changes were less extensive than those observed in the more basal regions.

In contrast, cochleae from SS-treated mice (Noise+SS) displayed remarkably well-preserved OHC architecture. DAPI staining (Fig. 5D) confirmed the preservation of OHC nuclei across all three cochlear turns, with no conspicuous nuclear loss even in the vulnerable medial and basal segments. Furthermore, phalloidin staining (Fig. 5C) showed distinct green fluorescence, revealing an intact OHC mosaic with orderly, regularly spaced stereocilia bundles that resembled the organization of the CTR group. The single row of IHCs was also maintained across all turns, further corroborating the protective effect of SS on the entire sensory epithelium against noise-induced trauma.

These structural findings are fully consistent with the functional ABR improvements — including preserved post-exposure thresholds and maintained Wave I amplitudes — described in Figure 4. Together, the convergent functional, biochemical, and histological evidence across Figs. 4 and 5 confirm that oral SS administration preserved cochlear sensory epithelial integrity across all turns in noise-exposed mice.

## Discussion

The present study provides a multi-tiered evaluation of SS extract as an otoprotective agent against oxidative stress–induced cochlear injury. Using complementary *in vitro*, ex vivo, and *in vivo* experimental models, we demonstrate that SS: (i) attenuates intracellular ROS accumulation and suppresses mitochondrial apoptotic signaling in UB-OC1 auditory cells; (ii) activates the Nrf2/HO-1 antioxidant signaling axis through enhanced Nrf2 nuclear translocation and consequent upregulation of HO-1 and NQO1; (iii) preserves cochlear hair cell structural integrity in organotypic explants under oxidative challenge; and (iv) maintains auditory function and suppresses cochlear lipid peroxidation in a murine model of noise-induced acoustic trauma. Collectively, these findings suggest that SS has potential as a functional ingredient to support cochlear health against oxidative stress in experimental models.

primary finding of this study is that SS effectively reduces intracellular ROS accumulation in oxidatively challenged UB-OC1 auditory cells, as demonstrated by two independent fluorometric assays — CellROX and DCFDA. The use of dual ROS detection methods provides complementary evidence of broad oxidative attenuation, as CellROX predominantly detects superoxide and hydroxyl radicals in the cytoplasm and mitochondria, while DCFDA reflects more broadly accumulated peroxides. The reduction in ROS burden by SS at concentrations of 100 and 200 μg/mL was comparable to that achieved by NAC, a well-characterized antioxidant used as a reference standard in NIHL research [30, 31]. This comparison is noteworthy because NAC exerts its antioxidant effect primarily through direct glutathione replenishment, a stoichiometric mechanism that does not inherently engage transcriptional antioxidant programs.

A mechanistically important distinction emerges from comparison of SS with NAC. While both agents achieved equivalent ROS suppression and anti-apoptotic efficacy (Figs. 1E–J), NAC failed to produce significant HO-1 protein upregulation (Fig. 2I–2J, ns), whereas SS dose-dependently and significantly induced HO-1 at all tested concentrations. This indicates that SS — unlike NAC — drives the complete Nrf2/HO-1 transcriptional cascade through to functional antioxidant gene induction, rather than merely initiating nuclear Nrf2 translocation. The hispidin validation data (Supplementary Figs. S2–S3) provide additional support for the Nrf2-activating capacity of an identified SS constituent [25, 26].

The suppression of mitochondrial apoptotic markers by SS provides important mechanistic context for its cytoprotective effects. The dose-dependent reductions in cleaved caspase-3 and cytochrome c observed across SS 50–200 μg/mL indicate inhibition of the intrinsic apoptotic cascade at multiple checkpoints: decreased cytochrome c release from the mitochondrial intermembrane space, and attenuated downstream activation of the executioner caspase-3. In contrast, p53 protein expression was not significantly altered by SS at any tested concentration, suggesting that SS does not primarily interfere with the p53-dependent transcriptional stress response, and that its anti-apoptotic effects are likely mediated downstream of p53 — possibly through direct attenuation of mitochondrial membrane permeabilization or modulation of Bcl-2 family proteins. Acoustic trauma has been established to activate both caspase-dependent and caspase-independent cell death pathways in cochlear hair cells [32, 33], and the targeted suppression of caspase-3 by SS therefore represents a mechanistically relevant protective strategy.

A key mechanistic insight from this study is the pattern of Nrf2 pathway activation induced by SS. ICC analysis revealed a dose-dependent increase in nuclear Nrf2 accumulation across all tested concentrations, accompanied by an increase in total Nrf2 protein, yet without a significant change in Keap1 protein levels at any concentration. The findings suggest that SS-derived bioactive components may promote Nrf2 nuclear translocation via Keap1-independent or Keap1-bypass mechanisms — for example, through direct protein-protein interaction interference, phosphorylation of Nrf2 by upstream kinases such as PI3K/Akt or MAPK/ERK that reduce Keap1 binding affinity, or through inhibition of the Cullin3-Keap1 E3 ubiquitin ligase complex that targets Nrf2 for proteasomal degradation [12, 13, 34]. Consistent with Nrf2 nuclear accumulation, HO-1 mRNA and protein were dose-dependently induced, confirming functional ARE-driven transcription. NQO1 was significantly induced only at the highest concentration (SS 200 μg/mL), while SOD1 was not significantly affected, indicating a differential threshold for ARE-driven gene activation.

The translation of cellular findings to ex vivo and *in vivo* models in this study was essential for establishing the biological relevance of SS. In the ex vivo organotypic explant system, SS conferred significant protection against H_2_O_2_-induced hair cell loss across all three tonotopic regions — apical, medial, and basal — with the degree of protection being statistically indistinguishable from the untreated control in all turns. This spatially comprehensive protection is noteworthy because previous studies have reported that antioxidant compounds often preferentially protect the more apically positioned, lower-frequency hair cells, while the metabolically vulnerable basal hair cells — which are preferentially destroyed in NIHL — remain more resistant to rescue [3]. The preservation of basal turn hair cells by SS in the *ex vivo* model, and the corresponding morphological preservation of OHC architecture across all turns *in vivo*, therefore suggests that SS may achieve a level of antioxidant amplification sufficient to protect even the most metabolically demanding cochlear cell populations. This is consistent with the hypothesis that Nrf2/HO-1 activation — which enhances cytoprotective gene expression rather than providing stoichiometric radical neutralization — is better suited than direct antioxidants to sustain protection under intense oxidative conditions [18].

In the *in vivo* acoustic stress model, ABR thresholds were assessed 24 h after noise exposure to capture early functional alterations directly linked to acute oxidative stress and the initiation of apoptotic signaling — the primary mechanistic target of Nrf2/HO-1-mediated otoprotection. Cochlear reactive oxygen species peak within the first 24–48 h following acoustic overexposure [1, 35, 36], and early ABR deficits at this timepoint have been shown to correlate with subsequent sensory cell pathology, including hair cell loss, in experimental NIHL models [34]. The preservation of ABR thresholds and Wave I amplitudes in SS-pretreated mice at 24 h therefore directly demonstrates reduced cochlear susceptibility to the acute oxidative apoptotic cascade most relevant to the cytoprotective mechanism under investigation. These findings represent robust and appropriately targeted *in vivo* evidence for the Nrf2/HO-1-mediated otoprotective mechanism. Longer-term ABR monitoring at 7–14 days post-exposure will be required to establish permanent threshold protection; such longitudinal studies would also allow evaluation of SS efficacy against progressive cochlear degeneration relevant to age-related hearing loss — a direction designated as a priority for future investigation.

Beyond threshold sensitivity, the preservation of ABR Wave I amplitudes in SS-treated mice across the full stimulus intensity range (20–90 dB SPL) carries additional mechanistic implications. ABR Wave I reflects the synchronous firing of auditory nerve fibers innervating IHCs, and is sensitive to both hair cell survival and IHC–SGN synaptic integrity. Noise-induced cochlear synaptopathy — the loss of ribbon synapses between IHCs and SGNs — is now recognized as an early form of cochlear pathology, often preceding hair cell loss [39–41]. The data suggest that SS may support synaptic integrity in addition to hair cell protection; direct quantification of synaptic ribbon density (CtBP2/GluA2 co-localization) in future experiments will be required to test this hypothesis.

The suppression of 4-HNE immunoreactivity in cochlear cross-sections from SS-treated mice provides direct biochemical evidence of reduced lipid peroxidation at the tissue level *in vivo*. 4-HNE is a highly reactive α,β-unsaturated aldehyde generated from the peroxidation of omega-6 polyunsaturated fatty acids — particularly arachidonic acid — and exerts cytotoxicity through the formation of protein adducts, DNA damage, and further amplification of the oxidative cascade [35]. Its accumulation in both IHC and OHC regions of noise-exposed cochleae is well-documented and directly correlates with sensory cell dysfunction [36]. The significant reduction of 4-HNE in SS-treated animals — despite exposure to identical acoustic trauma — provides convergent *in vivo* biochemical support for the Nrf2/HO-1 activation and ROS suppression observed in cellular experiments. HO-1, the most consistently and robustly induced Nrf2 target gene in our study, catalyzes the degradation of heme to produce carbon monoxide, free iron, and biliverdin — the latter of which exerts potent antioxidant and anti-lipid peroxidation activity — providing a plausible biochemical link between HO-1 induction and the suppression of 4-HNE accumulation observed *in vivo* [12].

From the perspective of functional food science and microbial biotechnology, this study highlights SS as a promising fungal-derived bioactive resource with potential application in developing value-added otoprotective functional materials. Despite extensive preclinical and clinical investigation of antioxidant strategies for NIHL prevention — including NAC, vitamins C and E, magnesium, and various polyphenolic compounds — no pharmacological agent has yet received regulatory approval for NIHL prevention [3]. This gap emphasizes the potential for exploring natural bioactive matrices as dietary options for supporting auditory well-being. SS has a long history of use in traditional East Asian medicine and has been evaluated in multiple preclinical models for hepatoprotective, immunomodulatory, and anti-tumor activities with an established safety profile [22, 23]. The 70% EtOH extraction yielded 9.97% (w/w) extract with consistent bioactivity across experimental systems. The *in vivo* dose of 200 mg/kg/day corresponds to an HED of approximately 16.2 mg/kg/day by body surface area normalization [44], within the 1–2 g/day range reported for SS in traditional and commercial use [33]. Bioavailability studies examining intestinal absorption, first-pass metabolism, and cochlear tissue distribution will be required to validate this translational relevance.

Several limitations of the present study should be acknowledged. First, the *in vivo* experiments utilized a relatively small sample size (n = 5–6 per group), which, while sufficient for detecting acute-phase functional and structural differences, may limit the detection of subtle long-term trends or strain-specific variability. Second, the C57BL/6 mouse strain harbors the Cdh23ahl (753A) variant, predisposing it to early-onset age-related hearing loss — a characteristic that provides a sensitive model for acoustic vulnerability but limits generalizability to genetically diverse auditory profiles; validation in additional strains (e.g., CBA/J or outbred strains) will be necessary. ABR measurements were performed at a single 24-h post-exposure timepoint, selected to capture the acute oxidative injury phase most relevant to Nrf2/HO-1-mediated otoprotection [1, 35, 36]; longitudinal ABR monitoring at 7–14 days post-noise will be required to establish permanent threshold protection and to evaluate potential SS efficacy against age-related cochlear degeneration. Fourth, while hispidin was evaluated as a representative identified SS constituent (Supplementary Figs. S2–3), the HPLC chromatogram contains additional uncharacterized peaks; the full mechanistic contributions and synergistic interactions of all SS constituents remain to be resolved through future bioactivity-guided fractionation. Fifth, the H_2_O_2_/UB-OC1 *in vitro* model does not fully replicate the complex *in vivo* cochlear microenvironment; the concordance between cellular and *in vivo* results nonetheless provides reassurance that the model captures relevant redox-responsive pathways.

In conclusion, this study provides multi-level experimental evidence that SS administration reduced oxidative damage markers within cochlear tissues of noise-exposed mice through active engagement of the Nrf2/HO-1 antioxidant signaling axis. The convergent cytoprotective, antioxidant, anti-apoptotic, and structural preservation effects observed across *in vitro*, *ex vivo*, and *in vivo* models, together with the *in vivo* suppression of 4-HNE-mediated lipid peroxidation and preservation of ABR function and cochlear architecture, collectively demonstrate the biological plausibility and functional relevance of SS as a candidate otoprotective bioactive material. From a microbiology and biotechnology standpoint, these findings position SSas a biologically productive fungal species whose secondary metabolite profile is well-suited to modulating cellular redox homeostasis in metabolically demanding sensory tissues. Further studies characterizing the active constituents of SS, their molecular targets within the Nrf2 regulatory network, and their pharmacokinetic behavior *in vivo* will be essential for supporting the use of SS-derived extracts as functional food ingredients for mitigating oxidative damage and supporting auditory well-being.

## Supplemental Materials

Supplementary data for this paper are available on-line only at http://jmb.or.kr.
