# Supplementary material for: A Fungal-Derived Bioactive Resource for Cochlear Protection: Sanghuangporus sanghuang Extract Mitigates Acoustic Trauma through Nrf2/HO-1 Antioxidant Axis
Source: J Microbiol Biotechnol. 2026 Jun 8;36:e2605011. doi: 10.4014/jmb.2605.05011 (PMC13265236; doi:10.4014/jmb.2605.05011)
Supplement: Supplementary file 1 [file jmb-36-e2605011-supple.pdf]

J. Microbiol. Biotechnol. 2026. 36: e2605011

https://doi.org/10.4014/jmb.2605.05011

A Fungal-Derived Bioactive Resource for Cochlear Protection: *Sanghuangporus sanghuang* Extract Mitigates Acoustic Trauma through Nrf2/HO-1 Antioxidant Axis

Hojin Lee[1,2]*, Changho Lee[1], Yun-Tai Kim[1], JaeYong Park[2] and Jaekwang Lee[1,3†]

[1] Food Functionality Research Division, Korea Food Research Institute, Wanju 55365, Korea

[2] School of Biosystems and Biomedical Sciences, College of Health Sciences, Korea University, Seoul 02841, Korea

[3] Major in Food Biotechnology, University of Science and Technology, Daejeon, 34113, Korea

† Correspondence to:

Jaekwang Lee, E-mail address: jklee@kfri.re.kr

Supplementary Figures and Table

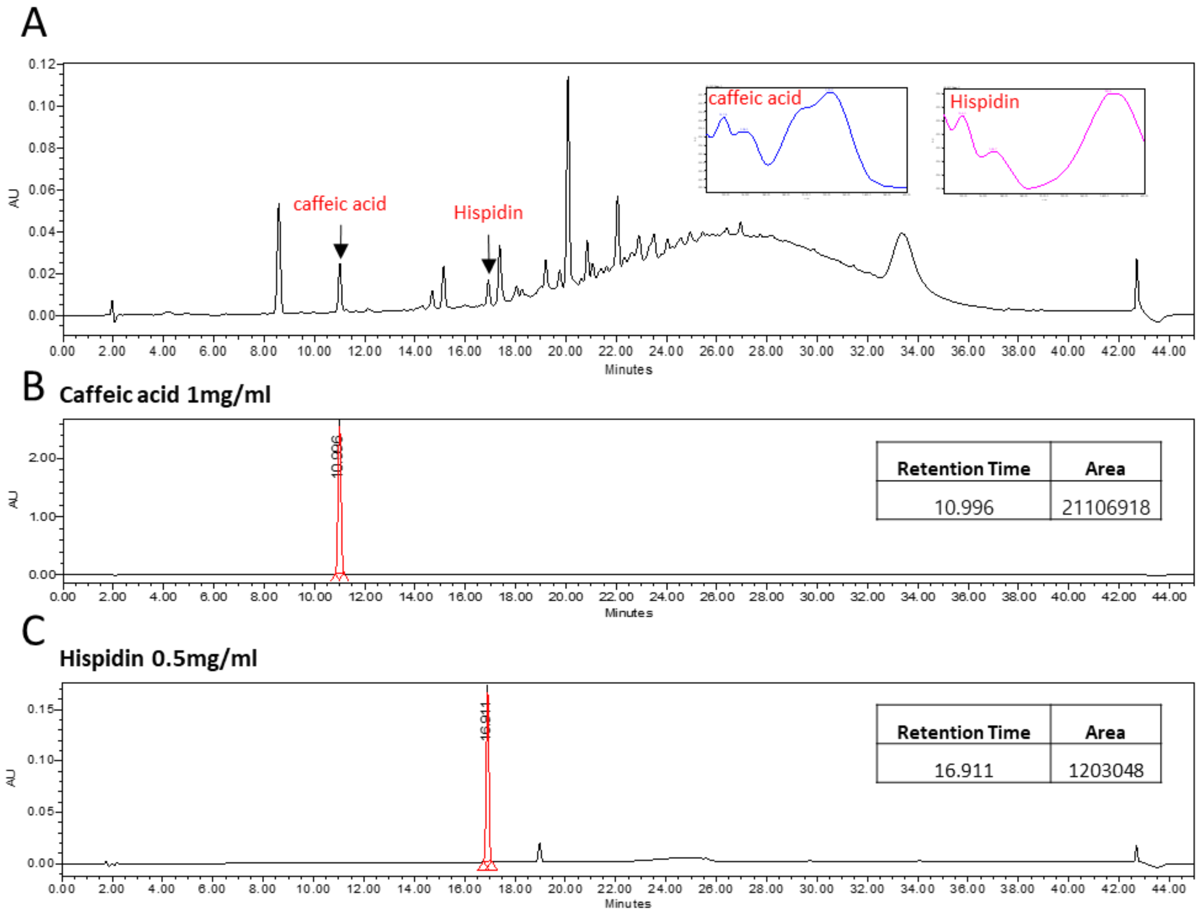

Fig. S1. HPLC chromatograms of *Sanghuangporus sanghuang* extract and reference compounds.

(A) HPLC profile of the 70% ethanol extract from *Sanghuangporus sanghuang*.

(B) HPLC peak of the caffeic acid standard.

(C) HPLC peak of the hispidin standard. The retention times (Rt) for each peak are indicated in the respective chromatograms.

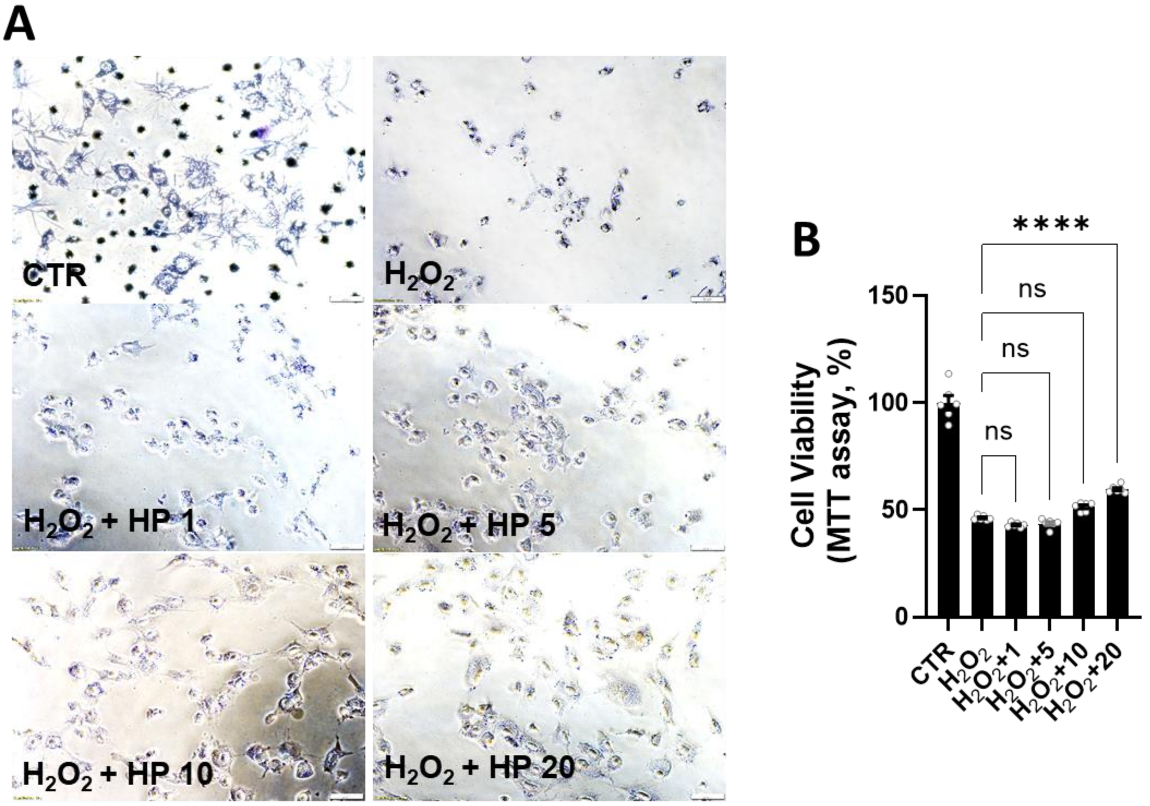

Fig. S2. Protective effect of hispidin against H_2O_2-induced cell death.

(A) MTT assay images of cells treated with hispidin (1–20 μM) and H_2O_2.

(B) Quantification of cell viability based on MTT assay absorbance.

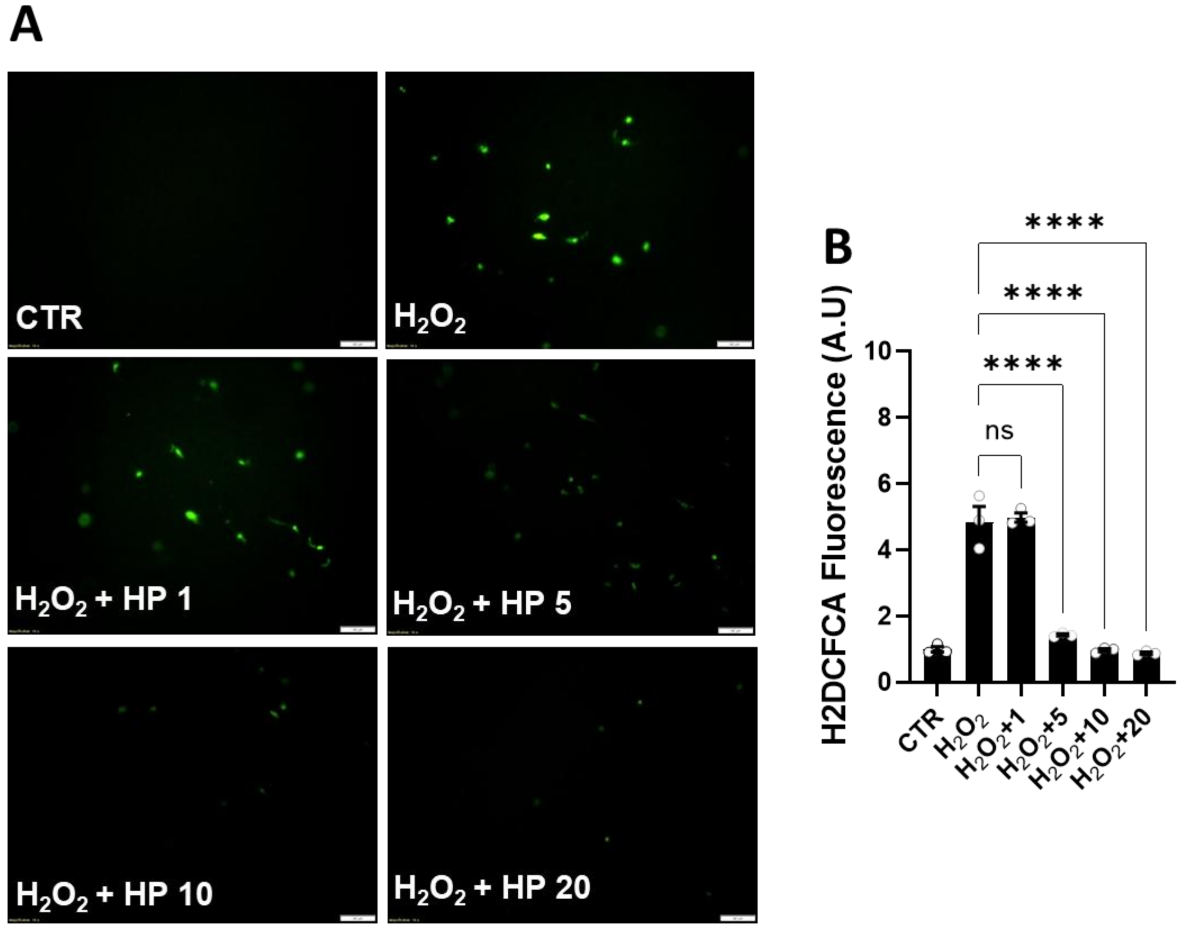

Fig. S3. Hispidin inhibits H_2O_2-induced ROS production.

(A) Representative H2DCFDA fluorescence images showing intracellular ROS levels. Cells were treated with hispidin (1–20 μM) and subsequent H_2O_2 exposure.

(B) Quantitative analysis of ROS production measured by H2DCFDA fluorescence intensity. Data are expressed as relative fluorescence intensity compared to the H_2O_2 group.

Table S1. Retention times of Compounds Used in the HPLC Analysis.

	Retention Time	Area
1	8.58	456969
2	11.011	165392
3	15.135	148125
4	16.914	79932
5	17.366	204299
6	19.199	78905
7	20.081	727906
8	20.849	114016
9	22.055	261682

	RT(time)	Area	Ditected
Hispidin	16.911	1203048	Yes
Hispolon	25.604	246543	ND
Protocatechuic	5.647	422774	ND
Galic acid	2.921	495968	ND
epicatechin	12.216	2745272	ND
Caffeic acid	10.996	21106918	Yes

*ND : not detected

Table S2.

Name	Manufacturer	Cat. No.	Dilution
4-HNE antibody	Abcam	ab48506	1:1000
Nrf2 antibody	CST	12721s,	1:500-1000
Keap1 antibody	Thermo Fisher	MA5-17106	1:500-1000
HO-1 antibody	Abcam	ab13243	1:1000
p53 antibody	CST	9282	1:1000
Cleaved Caspase-3 antibody	CST	9661	1:1000
Cytochrome c antibody	CST	4272	1:1000
β-Actin antibody	Santa Cruz	sc-47778	1:2000
Goat anti-Rabbit IgG (HRP)	Bio Rad	1706515	1:5000
Goat anti-mouse IgG (HRP)	Bio Rad	1706516	1:5000
Alexa Fluor 488 Phalloidin	Invitrogen	A12379	1:400
Goat anti-Rabbit IgG (H+L), Alexa Fluor 488	Abcam	ab150109	1:1000
Goat anti-Mouse IgG (H+L), Alexa Fluor 555	Abcam	ab150086	1:1000

MTT Reagent	Sigma-Aldrich	M2128	Cell Viability
CellROX™ Green Reagent	Invitrogen	C10444	ROS Assay
Fluoroshield™ with DAPI	Sigma-Aldrich	F6057	Mounting
OCT Compound	Sakura Finetek	4583	Cryosection
N-Acetyl-L-cysteine (NAC)	Sigma-Aldrich	A7250	Antioxidant
